# Phase II calm extension study: Coxsackievirus A21 delivered intratumorally to patients with advanced melanoma induces immune-cell infiltration in the tumor microenvironment

**DOI:** 10.1186/2051-1426-3-S2-P343

**Published:** 2015-11-04

**Authors:** Robert HI Andtbacka, Brendan D Curti, Sigrun Hallmeyer, Zipei Feng, Christopher Paustian, Carlo Bifulco, Bernard Fox, Mark Grose, Darren Shafren

**Affiliations:** 1 University of Utah Huntsman Cancer Institute, Salt Lake City, UT, USA; 2 Providence Cancer Center, Providence Portland Medical Center, Portland, OR, USA; 3 Oncology Specialsists, Park Ridge, IL, USA; 4 Earle A. Chiles Research Institute, Portland, OR, USA; 5 Chiles Research Institute, Providence Cancer Center, Portland, OR, USA; 6 Earle A. Chiles Research Institute, Providence Cancer Center, Portland, OR, USA; 7 Viralytics Limited, Toronto, ON, Canada; 8 Viralytics, Sydney, Australia

## Background

CAVATAK, an oncolytic immunotherapy, is a bio-selected oncolytic strain of Coxsackievirus A21 (CVA21). Following intratumoral (IT) injection, CVA21 preferentially infects ICAM-1 expressing tumor cells, resulting in viral replication, cell lysis, and a systemic anti-tumor immune response. The Phase II CALM study investigated the efficacy and safety of IT CVA21 in pts with advanced melanoma. The primary endpoint of the study was achieved with 22 of 57 (38.6%) evaluable pts with durable responses observed in both injected and uninjected melanoma metastases, suggesting the generation of significant host anti-tumor responses. Pre-clinical studies in an immune-competent mouse model of melanoma revealed that combinations of intratumoral CAVATAK and anti-PD-1 or anti-CTLA-4 mAbs mediated significantly greater anti-tumor activity compared to use of either agent alone. Here we report on a continuation study aimed at understanding the immune mediated effects of CVA21.

## Methods

To further elucidate the nature of the systemic anti-tumor responses, a CALM study (NCT01227551) extension cohort of 13 pts received up to 3 x 10^8^ TCID_50_ CVA21 IT on study days 1,3,5 and 8 and then every three weeks for a further 6 injections. Sequential tumor biopsies of both injected and uninjected lesions were monitored for levels of viral replication and evidence of viral-induced immune activation within the tumor micro-environment. Serial serum samples were monitored for viral loads, anti-CVA21 neutralizing antibody (nAb) and levels of immune-inflammatory cytokines.

## Results

CVA21 administration was shown in 5 of 6 cases to induce increases in immune cell infiltrates within the tumor microenvironment, in particular CD8^+^cells and increased expression of PD-L1^+^ cells as assessed by multispectral imaging. Reconstitution of immune cell infiltrates was observed in 4 of 4 of CVA21 treated lesions from patients failing treatment with single or double-line immune-checkpoint blockade. Analysis of 4 pre- and post-treatment biopsy samples by NanoString digital RNA counting identified sizable up-regulation of a number of immune modulation elements, including a Th1-gene shift, with increases in interferon-induced genes.

## Conclusions

Intralesional administration of CVA21 can notably influence the dynamics of the tumor micro-environment as evidenced by increases in immune cell infiltrates and immune-related response genes. Our observation that CVA21 can reconstitute immune cell infiltrates in lesions resistant to immune-checkpoint blockade provides a strong rationale for the investigation of the sequential or concurrent administration of CVA21 and T cell checkpoint antibodies. A clinical trial combining CVA21 with anti-CTLA-4 is underway and other combination studies are planned.

